# Investigating the Bidirectional Associations of Adiposity with Sleep Duration in Older Adults: The English Longitudinal Study of Ageing (ELSA)

**DOI:** 10.1038/srep40250

**Published:** 2017-01-09

**Authors:** Victoria Garfield, Clare H. Llewellyn, Andrew Steptoe, Meena Kumari

**Affiliations:** 1 Department of Epidemiology & Public Health, University College London, UK; 2 Institute for Social & Economic Research, University of Essex, UK.

## Abstract

Cross-sectional analyses of adiposity and sleep duration in younger adults suggest that increased adiposity is associated with shorter sleep. Prospective studies have yielded mixed findings, and the direction of this association in older adults is unclear. We examined the cross-sectional and potential bi-directional, prospective associations between adiposity and sleep duration (covariates included demographics, health behaviours, and health problems) in 5,015 respondents from the English Longitudinal Study of Ageing (ELSA), at baseline and follow-up. Following adjustment for covariates, we observed no significant cross-sectional relationship between body mass index (BMI) and sleep duration [(unstandardized) B = −0.28 minutes, (95% Confidence Intervals (CI) = −0.012; 0.002), *p* = 0.190], or waist circumference (WC) and sleep duration [(unstandardized) B = −0.10 minutes, (95% CI = −0.004; 0.001), *p* = 0.270]. Prospectively, both baseline BMI [B = −0.42 minutes, (95% CI = −0.013; −0.002), *p* = 0.013] and WC [B = −0.18 minutes, (95% CI = −0.005; −0.000), *p* = 0.016] were associated with decreased sleep duration at follow-up, independently of covariates. There was, however, no association between baseline sleep duration and change in BMI or WC (*p* > 0.05). In older adults, our findings suggested that greater adiposity is associated with decreases in sleep duration over time; however the effect was very small.

Obesity is associated with adverse physical^1^,^2^,^3^ as well as psychological/psychiatric health outcomes^4^,^5^,^6^,^7^,^8^. Poor sleep is associated with cardiovascular disease^9^ and this could be due to adiposity. However, conclusive studies of the association of sleep and adiposity are predominantly limited to cross-sectional studies, while prospective data have yielded mixed findings, particularly in older adults.

In younger adults cross-sectional data have consistently found that self-reported short sleep is independently associated with greater weight, as reported in two systematic reviews^10^,^11^. Cross-sectional data also suggest that the association of self-reported sleep measures and body weight can be non-linear^12^, such that both under- and overweight are associated with shorter sleep duration. The evidence for this association in older adults is, however, less consistent than in younger adults^13^,^14^,^15^ with studies that suggest no association^14^ or associations that are similar in magnitude to those seen in younger age groups^13^,^15^,^16^,^17^.

Prospectively, while there is some evidence for an association between weight change and change in sleep duration in adults, associations are mixed and may vary by age group^10^,^11^,^16^,^18^. A review of three prospective studies suggests a relation between short sleep and future weight gain^11^. In younger adults shorter sleep was found to be associated with weight gain in the Zurich Cohort Study^16^, the NHANES study^18^, the Nurses’ Health Study^19^ and in the Quebec Family Study^12^. However, measures of sleep duration failed to predict changes in BMI in the Coronary Artery Risk in Young Adults (CARDIA) Sleep Study^20^ and in 1 648 Japanese men aged between nineteen and thirty-nine years^21^.

Research also suggests that, in addition to an association of short sleep and increased BMI, long sleep duration, defined as 9 to 10 hours may also increase the risk of weight gain in adults^12^. Evidence has also emerged in favour of a longitudinal association between long sleep and weight loss^16^. Few studies have examined the longitudinal relationship of sleep duration and BMI in older adults and have also yielded ambiguous results. The Whitehall II study found no evidence of an association between shorter sleep duration and changes in BMI or obesity incidence over 4 years, in a sample with a mean age of fifty-six^22^. However, prospective analysis of 3 576 Spanish older adults suggests that a sleep duration of less than or equal to 5 hours, as well as a sleep duration of 8 or 9 hours is associated with obesity; and with weight gain over a period of two years, but only in females^13^.

As far as we can determine, no studies have to date, tested the prospective, bidirectional relation between BMI and sleep duration in a single study, particularly in an ageing community sample. This is important, as it has been suggested that this association could be bidirectional in nature^23^, such that greater BMI may precede shorter sleep duration^12^,^15^,^20^ or shorter sleep duration may precede weight gain^12^,^15^,^20^. It is essential to ascertain the direction of this association to allow health professionals to better understand where to target interventions in older age groups, when disease and frailty are most likely to occur.

However, the use of BMI as an adiposity indicator in older adults may not be optimal, due to its reduced ability to predict body fat in this population, due to decreasing muscle mass with age (e.g. sarcopenia)^24^,^25^. Few studies have investigated the association of alternative adiposity measures with sleep duration, such as waist circumference (WC), with evidence emerging in favour of a cross-sectional^26^,^27^, but not a prospective relation^22^,^28^. No studies have yet examined the association between WC and potential change in sleep duration over time, nor have they incorporated bidirectional analyses.

Due to these shortcomings in the current literature, we sought to investigate the bidirectional association between BMI and sleep duration, as well as WC and sleep duration, in a large, nationally representative, ageing sample, which is rich in covariates and prospective data. Here we present findings from cross-sectional and bidirectional prospective associations between BMI and sleep duration, and waist circumference and sleep duration, in the English Longitudinal Study of Ageing (ELSA), with the inclusion of a wide range of covariates, which may affect this relationship. Our aims were twofold: i) to examine the cross-sectional relationships of BMI and waist circumference with sleep duration in older adults and ii) to ascertain the nature of the direction of this association, using longitudinal data to examine the association from baseline BMI and WC, to change in sleep duration over a 4-year period, and baseline sleep duration to change in BMI and WC, from baseline to follow-up.

## Methods

### Sample

#### English Longitudinal Study of Ageing (ELSA)

Data are used from the English Longitudinal Study of Ageing (ELSA), which is an on-going national panel study of health and ageing initiated in 2002–3 (wave 1). Data have been collected from respondents at waves 2 (2004–5), 3 (2006–7), 4 (2008–9), 5 (2010–11) and 6 (2012–13), and comprise a nationally representative sample of English household residents aged fifty and over. Further details of ELSA can be found elsewhere^29^. ELSA was granted ethical approval by the London Multicentre Research Ethics Committee (MREC 01/2/91) and all participants provide informed consent at each wave. All methods were carried out in accordance with approved guidelines and regulations.

Of the 11,050 ELSA respondents at wave 4, 8,210 were interviewed and clinical measurements made by a nurse; whilst at wave 6 there were 10,601 interviews and 7,731 nurse visits. We analysed data from 5,015 respondents from waves 4 and 6 of ELSA; inclusion of respondents was based on whether they had complete data for measures of adiposity, sleep duration and all covariates at both waves of data collection.

### Measures

#### Body Mass Index

Participants were visited in their home by a nurse who measured both height and weight at waves 4 and 6. Standing height was measured using a Leicester portable stadiometer standardized with head in the Frankfort plane. A single weight measurement was recorded to the nearest 0.1 kg, using Tanita THD 305 scales. BMI was subsequently derived using the standard formula: weight divided by height squared (kg/m^2^).

#### Waist circumference

During the nurse visit, two measurements of waist circumference were taken at the midpoint between the lower rib and the iliac crest using measuring tape. A mean of the two measurements was then used, unless they differed by more than 3 cm, in which case a third measurement was taken and then an average of the closest two measurements was used.

#### Sleep duration and change in sleep duration

A question on sleep duration was included in ELSA for the first time at wave 4 and repeated at wave 6. Respondents were asked ‘How many hours of sleep do you have on an average week night?’ Change in sleep duration from baseline to follow-up was calculated by subtracting sleep duration at wave 6 from sleep duration at wave 4.

#### Covariates

Demographic, socio-economic and health behaviour measures collected at wave 4 were used as covariates in the analyses. Age was recorded as a continuous number until 90 years, with ages above 90 collapsed to the value of 91. Socio-economic position was determined by quintiles of non-pension wealth, which is regarded as the most salient measure of standard of living in older age groups^30^. Frequency of alcohol consumption within the last 12 months [categorised to less than daily; daily (5–7 times per week)]; smoking status (never; ex-smoker; current smoker); long-standing illness (respondents were asked: ‘do you have any long-standing illness, disability or infirmity? by long-standing I mean anything that has troubled over a period of time, or that is likely to affect over a period of time’, to which they could answer ‘No’ or ‘Yes’); physical activity levels (Sedentary; Low; Moderate; High), and depressive symptoms (measured with the Centre for Epidemiologic Studies – Depression Scale – CESD – 8 item scale) were also assessed by questionnaire. CES-D responses were summed (with the exception of the item: “whether respondent felt their sleep was restless in the past week”) to obtain a total score, which was then dichotomized using a cut-off of > = 3^31^. A dichotomous season variable was created using the date which respondents completed their interview, which resulted in a categorisation of 0 = “BST” (British Summer Time) and 1 = “GMT” (Greenwich Mean Time). However, no association was observed between season at baseline and sleep duration at wave 4 [β = 0.022, (95% CI = −0.047; 0.091), *P* = 0.533] or wave 6 [β = 0.009, (95% CI = −0.079; 0.061), *P* = 0.800] when adjusting for age and sex, and was therefore not included in subsequent analyses.

### Statistical analyses

All analyses were performed in STATA, version 13. Pearson’s correlations were used to examine the relationship between sleep duration at baseline (wave 4) and follow-up (wave 6). For examination of baseline sample characteristics sleep categories of ≤5 hours, 6–7 hours, 7–8 hours and >9 hours (Table 1.) were created. Analysis of variance was used to compare means for age, BMI and WC, whilst chi-squared tests were used to examine differences in categorical demographic variables (smoking status, alcohol consumption, long-standing illness, wealth, sex, ethnicity and depressive symptoms) across the 4 sleep duration groups.

Initially, quadratic regression modelling was used to investigate potential non-linear associations in cross-sectional and prospective, bidirectional relationships between adiposity measures (BMI and WC) and sleep duration. Multicollinearity was tested in all regression models using the variance inflation factor (VIF) to examine the extent to which predictors were correlated. A VIF of 1 indicates no correlation, whilst values >10 are generally cause for concern^32^.

For cross-sectional analyses, 4 regression models were performed to examine the association between BMI and sleep duration, with the same models run to investigate the relation between WC and sleep duration. Model 1 was minimally-adjusted (age, sex, wealth, ethnicity), whilst models 2 and 3 were adjusted for health behaviours (minimally-adjusted + alcohol consumption, smoking status, physical activity levels) and health problems (minimally-adjusted + depressive symptoms, long-standing illness), respectively and model 4 was fully-adjusted for all covariates (minimally-adjusted + health behaviours + health problems).

Prospectively, the associations between adiposity (BMI and WC) and sleep duration, and sleep duration and adiposity were investigated using both linear and quadratic models. In order to examine change in sleep duration, sleep duration at wave 6 was analysed as the outcome with BMI or WC at baseline adjusted for sleep duration at baseline as the exposure. Conversely, in analyses to examine changes in BMI and WC, BMI or WC at follow-up was analysed as the outcome with sleep duration at baseline adjusted for BMI or WC at baseline as the exposure. Aside from this difference, Models 1 to 4 were identical to the cross-sectional models described above.

In order to examine the role of covariates in the association of adiposity measures and sleep duration, the percentage reduction in the regression coefficient following adjustment was calculated by comparing the coefficient for each exposure from models with and without adjustment for covariates.

## Results

Compared with all participants at wave 4 of ELSA, those included in the present analyses were wealthier, slightly older and less likely to report having a long-standing illness (all p < 0.001).

Table 1 shows baseline (wave 4) characteristics of participants, according to their sleep duration category. One-way ANOVAs showed that there were significant differences across sleep duration categories for both age and BMI, such that younger respondents slept for six to seven hours, whilst the oldest group slept, on average, for nine or more hours; the heaviest respondents had mean sleep durations of less than, or equal to five hours. There were, however, no significant differences in baseline waist circumference across the four sleep duration categories.

Chi-squared analyses showed that sex, smoking status, alcohol consumption, limiting illness, wealth, depressive symptoms and physical activity levels, were all significantly associated with sleep duration. Short sleepers (≤5 hours) were significantly more likely to be females, ex-smokers, less wealthy, consume less alcohol, report a long-standing illness and engage in ‘moderate’ physical activity (Table 1). Multicollinearity was not an issue in any of our cross-sectional or prospective regression models, as all VIF values were around 1 when tested.

Mean BMIs were 28.20 kg/m^2^ and 28.17 kg/m^2^, at baseline and follow-up respectively; whilst average duration of sleep was 6.86 hours at baseline and 6.87 hours at follow-up. Respondents who slept for five hours or less had the highest mean BMI (28.89 kg/m^2^) both at baseline and follow-up, whilst those who slept between eight and nine hours had the lowest mean BMI (28.06 kg/m^2^). Overall mean WC at baseline was 96.49 cm and 96.09 cm at follow-up.

### Cross-sectional associations: BMI and sleep duration at baseline; WC and sleep duration at baseline

A basic model revealed a small, inverse linear relation between BMI and sleep duration, which was attenuated and no longer significant in model 2 adjusted for health behaviours (basic model to health-behaviours adjusted model = 5% decrease in the coefficient), and then further weakened in model 3 adjusted for health problems (basic model to model adjusted for health problems = 15% decrease in the coefficient). In the final model adjusted for all covariates this effect was again, attenuated (basic model to final model = 16% decrease in the coefficient) (Table 2). There was no interaction between sex (p = 0.822) or age (p = 0.366) and BMI at baseline on sleep duration, in any of the four regression models. We also performed quadratic cross-sectional regression models to test for a U-shaped relationship between BMI and sleep duration, but this was not significant (p = 0.987).

The pattern of results for waist circumference and sleep duration was almost identical to that of BMI and sleep duration (Table 2). In a model adjusted only for demographics there was a significant, negative association between WC and sleep duration, which was attenuated with inclusion of health behaviours in Model 2 (basic model to model adjusted for health behaviours = 4% decrease in the coefficient). With adjustments for health problems, the coefficient was again, reduced (basic model to health problems-adjusted model = 7% decrease in the coefficient) and a final model including all covariates resulted in further attenuation (basic model to final model = 8% decrease in the coefficient). We observed no evidence of a U-shaped association between baseline WC and sleep duration (p = 0.103), nor did we find a significant interaction of age (p = 0.084), or sex (p = 0.300) with baseline WC on sleep duration.

### Prospective associations I: BMI and changes in sleep duration; WC and changes in sleep duration

The first set of prospective analyses performed had baseline BMI as the exposure and follow-up sleep duration as the outcome, the results of which are shown in Table 3. Model 1 revealed a negative association between baseline BMI and follow-up sleep duration, such that a higher BMI was associated with increasingly shorter sleep at wave 6. In Model 4, the longitudinal association between BMI and sleep duration was only slightly attenuated (Model 1 to Model 4, 6% non-significant decrease in the coefficient, p > 0.05). On average, the change in sleep duration from baseline to follow-up was −0.42 minutes per unit increase in BMI. A very similar pattern of associations was observed between WC and changes in sleep duration, such that for every centimetre increase in WC at baseline, sleep duration at follow up decreased, on average, by 0.18 minutes. Although small, this effect remained significant after adjustment for all covariates in Model 4 and the coefficient was identical throughout the models (Table 3). The mean change in sleep duration from baseline (6.867 hours) to follow-up (6.872 hours) was 0.005 hours (0.3 minutes), standard deviation = 1.13 hours (67.5 minutes).

There were no interactions between baseline age or sex with BMI and WC on follow-up sleep duration in any of the 4 models (p > 0.05). There was no evidence of a quadratic association between baseline BMI or WC and follow-up sleep duration (p > 0.05).

### Prospective associations II: Sleep duration and changes in BMI; sleep duration and changes in WC

Conversely, the longitudinal analyses presented in Table 4 revealed no significant associations between sleep duration and future BMI. Across all 4 models, there was no evidence of a linear association between sleep duration at baseline and BMI at 4-year follow-up. Nor was a U-shaped association observed (p > 0.05). Although age at baseline was strongly associated with BMI at follow-up [B = −0.020 hours, (95% CI = −0.027; −0.013) *P* < 0.001] after adjustment for BMI at baseline and all other covariates, there were no interactions between baseline age or sex with sleep duration on BMI at follow-up in any of the 4 models (all p > 0.05). Similarly to sleep duration and changes in BMI, there was no significant association between sleep duration at baseline and changes in waist circumference (Table 4) in any of our 4 regression models (all p > 0.05). There were also no significant interactions between age and baseline sleep duration, or sex and baseline sleep duration on follow-up WC, nor was there evidence of a quadratic association (all p > 0.05).

## Discussion

In this large, nationally representative study of older adults, findings suggest that cross-sectionally, while both BMI and WC are inversely associated with sleep duration, these relationships are largely accounted for by variations in health status and health behaviours. Prospectively, greater BMI and WC at baseline were associated with small decreases in sleep duration over a 4-year period, independently of adjustment for a variety of covariates. In contrast, sleep duration at baseline was not associated with changes in BMI or WC over the follow-up period.

When tested we found no statistically significant evidence of a cross-sectional U-shaped relationship between BMI and sleep duration. The findings of longest sleep duration in those with BMI between 18.5 and 24.9 kg/m^2^ agrees with previous research^10^,^11^,^20^,^33^,^34^. This may reflect reverse causation, as in older age groups long-standing illness is prevalent and may lead to weight loss. Our findings support this notion because adjustment for health problems attenuated observed associations. It is also important to note that, for example, the BMI and WC of a respondent with diabetes could be quite different from a respondent reporting cancer.

Our findings indicate that BMI and WC are not independently associated with sleep duration cross-sectionally, a result consistent with two earlier large-scale studies, which did not find an association between adiposity and sleep duration^18^,^35^. However, our results do not accord with recent evidence in favour of this cross-sectional relationship in older adults^13^,^15^,^36^. At least one of these studies, which found a significant association of BMI and WC with sleep duration in older adults made no adjustment for physical long-standing illness or socioeconomic position in their analysis^15^, which could in part explain the discrepancy between our findings and theirs. These authors also used a measure of self-reported sleep duration by which respondents were only asked to report how many hours they had slept on the two nights prior to the interview^15^ rather than the more general sleep duration question in ELSA which asked about the number of hours sleep on an average weeknight.

The richness of the available dataset enabled analyses that accounted for a number of factors, including wealth, illness and depressive symptoms, and health behaviours. Associations apparent in our data concur with several reports that associations exist between disadvantaged socioeconomic position and sleep duration^37^,^38^,^39^,^40^,^41^, and depression and sleep duration^42^,^43^. There is also evidence for an association between socioeconomic position and obesity^44^,^45^, as well as BMI and depression^46^. Thus, in future it would be of interest to further explore the interrelationship between BMI, these measures and sleep duration, particularly depression which is closely related to sleep behaviours, using measures taken at several time points.

Longitudinal analyses of adiposity at baseline with sleep duration at follow-up revealed a negative association, such that higher BMI and WC were associated with decreased length of sleep. However, in ELSA there was no evidence of an association between sleep duration and change in BMI from baseline to follow-up, nor between baseline sleep duration and change in WC at follow-up.

Finding that measures of adiposity and sleep duration were associated in a prospective analysis accords with previous research, which found evidence of a trend – albeit not statistically significant – for an association between average changes in weight gain and average change rates in sleep duration^16^,^18^. The present study found that both BMI and WC were associated with future sleep duration in a sample whose average age was 65 years. This result is in line with a study in younger adults, which suggests the association between adiposity and changes in sleep duration was stronger than the opposite relationship, which examined sleep duration and changes in adiposity^16^.

One plausible explanation for the prospective associations of adiposity measures and sleep duration in older adults could be obstructive sleep apnoea (OSA), a condition that causes the airways to collapse or become blocked whilst sleeping and is markedly prevalent in obese adults^47^. Older people with higher BMIs and/or waist circumferences may have a higher percentage of visceral fat than their leaner counterparts, which has been found to be a significant risk factor for OSA^47^,^48^. Therefore they may develop OSA, which could subsequently affect their sleep duration. Evidence suggests that when objectively measuring sleep duration, very short sleep (mean duration of 3 hours) is associated with greater OSA severity^49^, which could also be applicable to self-reported sleep duration. Another recent study found self-reported short sleep duration and OSA to be independently associated with visceral obesity, in adults aged between forty and sixty-nine years^50^.

Both body mass index and waist circumference remained associated with change in sleep independently of a wide range of covariates, including health and health behaviours. However, we cannot discount the possibility of residual confounding, as it was not possible to examine all other factors that might explain the observed association between adiposity and sleep duration.

Our observation that sleep duration was not associated with change in BMI or WC specifically in older adults, accords with some^22^,^51^, but not all previous reports^13^. One potential explanation for this may relate to the stability of BMI, as neither average BMI or WC changed greatly in 4 years. Further follow-up of the participants may reveal associations not yet apparent. Secondly, it is suggested that the magnitude of the association between sleep duration and changes in adiposity measures declines with age^14^,^16^,^52^. This may explain why the results presented here, where mean age is 65, and in other studies such as Whitehall II^22^ are null. Thus, these data suggest that obesity may be a target to ameliorate co-morbidities that occur due to poor sleep, but that sleep duration is not a target to prevent obesity in older age groups.

Our study has a number of strengths. The longitudinal design of ELSA enabled us to investigate the bidirectional association between two measures of adiposity (BMI, WC) and sleep duration within the same population. The sample size was sufficient to observe an association between BMI and WC, and change in sleep duration. However, we failed to observe an association between sleep duration and change in adiposity measures, indicating that this association is likely to be weak in comparison. Also, BMI and WC were measured by a nurse, rather than self-reported, unlike some earlier studies in this area^33^,^53^. A further strength is that ELSA is broadly representative of the English population aged 50 years and older^29^.

Sleep duration was, however, self-reported which may be prone to error and bias^54^. Data were only available from waves 4 and 6 of ELSA; hence there were only four years between baseline and follow-up, which may have contributed to the trivial change we observed in sleep duration. This could perhaps be related to findings that usual sleep parameters do not significantly change in adults after the age of sixty^55^. We were also unable to examine potential mediators of the prospective association between adiposity measures and sleep duration. For example, respondents might sleep poorly due to their own or their partner’s snoring or other symptoms of sleep apnoea, as mentioned above. Additionally, information on daytime napping or shift work was not available, which is particularly pertinent in older adults.

In conclusion, we found that in older adults, BMI and waist circumference were associated with change in sleep duration from baseline to follow-up, rather than *vice versa*. This is important so that interventions can be targeted appropriately in older adults.

## Additional Information

**How to cite this article:** Garfield, V. *et al*. Investigating the Bidirectional Associations of Adiposity with Sleep Duration in Older Adults: The English Longitudinal Study of Ageing (ELSA). *Sci. Rep.* **7**, 40250; doi: 10.1038/srep40250 (2017).

**Publisher's note:** Springer Nature remains neutral with regard to jurisdictional claims in published maps and institutional affiliations.

## Figures and Tables

**Table 1:** Sample characteristics at baseline by sleep duration category (N = 5,015).

	≤5 hrs	6–7 hrs	8–9 hrs	>9 hrs	Total	*P*
	n = 632	n = 2,661	n = 1,661	n = 61	N = 5,015	
Age (years)**	65.25 (8.56)	64.29 (8.43)	65.15 (8.03)	68.51 (8.86)		<0.001
Sex**						<0.001
*Male*	219 (34.65)	1,243 (46.71)	755 (45.45)	21 (35.43)	2,238 (44.63)	
*Female*	413 (65.35)	1,418 (53.29)	906 (54.55)	40 (65.57)	2,777 (55.37)	
Ethnicity						>0.05
*White*	616 (97.47)	2,615 (98.27)	1,635 (98.43)	60 (98.36)	4,926 (98.23)	
*Non-white*	16 (2.53)	46 (1.73)	26 (1.57)	1 (1.64)	89 (1.77)	
BMI (kg/m^2^)**	28.75 (28.75)	28.10 (28.10)	27.99 (4.78)	28.09 (5.76)	5,015	<0.05
Waist circumference (cm)	97.02 (14.04)	96.5 (13.15)	96.13 (12.94)	97.15 (14.60)		>0.05
Smoking status*						<0.05
*Never smoked*	244 (38.61)	1,160 (43.59)	739 (44.49)	26 (42.62)	2,169 (43.25)	
*Ex-smoker*	290 (45.89)	1,198 (45.02)	751 (45.21)	23 (37.70)	2,262 (45.10)	
*Current smoker*	98 (15.51)	303 (11.39)	171 (10.30)	12 (19.67)	584 (11.65)	
Frequency of alcohol consumption*						<0.05
*Less than daily*	517 (81.80)	2,042 (76.74)	1,257 (75.68)	47 (77.05)	3,863 (77.03)	
*Daily (5–7 days per week)*	115 (18.20)	619 (23.26)	404 (24.32)	14 (22.95)	1,152 (22.97)	
Wealth quintile**						<0.001
*Lowest*	154 (24.37)	313 (11.76)	166 (9.99)	13 (21.31)	646 (12.88)	
*Others*	478 (75.63)	2,348 (88.24)	1 518 (90.01)	48 (78.69)	4,369 (87.12)	
Long-standing illness**						<0.001
*No*	246 (38.92)	1,323 (49.72)	842 (50.69)	20 (32.79)	2,431 (48.47)	
*Yes*	386 (61.08)	1,338 (50.28)	819 (49.31)	41 (67.21)	2,584 (51.53)	
CES-D depressive symptoms**						<0.001
*No*	293 (46.36)	1,859 (69.86)	1,221 (73.51)	32 (52.46)	3,405 (67.90)	
*Yes (score* > = *3)*	339 (53.64)	802 (30.14)	440 (26.49)	29 (47.54)	1,610 (32.10)	
Physical activity levels**						<0.05
*Sedentary*	36 (5.70)	64 (2.41)	36 (2.17)	4 (6.56)	140 (2.79)	
*Low*	185 (29.27)	497 (18.68)	285 (17.16)	22 (36.07)	989 (19.72)	
*Moderate*	291 (46.04)	1,440 (54.11)	956 (57.56)	22 (36/07)	2,709 (54.02)	
*High*	120 (18.99)	660 (24.80)	384 (23.12)	13 (21.31)	1,177 (23.47)	

BMI = Body Mass Index; WC = waist circumference, Means (SDs) or n (%).

**Table 2:** Cross-sectional associations between adiposity and sleep duration at Wave 4 of the English Longitudinal Study of Ageing (N = 5,015).

Sleep duration	B (minutes)	95% CI	P
**BMI**			
Basic model (1)	−0.44	−0.014–−0.000	0.033
Adjusted for health behaviours (2)	−0.39	−0.013–−0.000	0.065
Adjusted for health problems (3)	−0.29	−0.012–−0.002	0.167
Fully-adjusted model (4)	−0.28	−0.012–0.002	0.190
**Waist circumference**			
Basic model (1)	−0.18	−0.006–−0.000	0.034
Adjusted for health behaviours (2)	−0.14	−0.005–0.000	0.087
Adjusted for health problems (3)	−0.11	−0.005–−0.000	0.198
Fully-adjusted model (4)	−0.10	−0.004–0.001	0.270

(1) Adjusted for age, sex, wealth, ethnicity; (2) Basic model + physical activity, smoking status, alcohol consumption; (3) Basic model + long-standing illness, depressive symptoms; (4) Basic model + health behaviours + health; B (Unstandardized coefficient) = difference in sleep duration (minutes) per difference in WC (cm), 95% CI = 95% confidence interval, P = regression p-value.

**Table 3:** Prospective associations between adiposity at wave 4 and sleep duration at wave 6 of the English Longitudinal Study of Ageing (N = 5,015).

Sleep duration	B (minutes)	95% CI	P
**BMI**			
Basic model (1)	−0.48	−0.014–−0.003	0.004
Adjusted for health behaviours (2)	−0.48	−0.014–−0.003	0.004
Adjusted for health problems (3)	−0.48	−0.013–−0.002	0.012
Fully-adjusted model (4)	−0.42	−0.013–−0.002	0.013
**Waist circumference**			
Basic model (1)	−0.18	−0.006–−0.000	0.005
Adjusted for health behaviours (2)	−0.18	−0.006–−0.000	0.007
Adjusted for health problems (3)	−0.18	−0.005–−0.000	0.015
Fully-adjusted model (4)	−0.18	−0.005–−0.000	0.016

(1) Adjusted for age, sex, wealth, ethnicity and baseline sleep duration; (2) Basic model + physical activity, smoking status, alcohol consumption, baseline sleep duration; (3) Basic model + long-standing illness, depressive symptoms, baseline sleep duration; (4) Basic model + physical activity, smoking status, alcohol consumption, long-standing illness, depressive symptoms, baseline sleep duration; B (Unstandardized coefficient) = change in sleep duration (minutes) per unit change in BMI (kg/m^2^) or WC (cm), 95% CI = 95% confidence interval, P = regression p-value.

**Table 4:** Prospective associations between sleep duration at Wave 4 and adiposity at Wave 6 of the English Longitudinal Study of Ageing (N = 5,015).

Sleep duration			
BMI	B (kg/m^2^)	95% CI	P
Basic model (1)	0.005	−0.046–0.046	0.997
Adjusted for health behaviours (2)	0.042	−0.045–0.047	0.977
Adjusted for health problems (3)	0.11	−0.044–0.048	0.938
Fully-adjusted model (4)	0.12	−0.044–0.048	0.928
**Waist circumference**	**B (cm)**	**95% CI**	**P**
Basic model (1)	−0.06	−0.195–0.082	0.426
Adjusted for health behaviours (2)	−0.05	−0.189–0.089	0.480
Adjusted for health problems (3)	−0.06	−0.197–0.082	0.416
Fully-adjusted model (4)	−0.05	−0.194–0.085	0.447

(1) Adjusted for age, sex, wealth and baseline BMI or WC; (2) adjusted for age, sex, physical activity, smoking status, alcohol consumption, baseline BMI or WC; (3) adjusted for age, sex, long-standing illness, depressive symptoms, baseline BMI or WC; (4) adjusted for age, sex, wealth, physical activity, smoking status, alcohol consumption, long-standing illness, depressive symptoms, baseline BMI or WC; B (Unstandardized coefficient) = change in BMI (kg/m^2^) or WC (cm) per change in sleep duration (minutes), 95% CI = 95% confidence interval, P = regression p-value.
